# Altered glymphatic enhancement of cerebrospinal fluid tracer in individuals with chronic poor sleep quality

**DOI:** 10.1177/0271678X221090747

**Published:** 2022-03-29

**Authors:** Per Kristian Eide, Are Hugo Pripp, Benedikte Berge, Harald Hrubos-Strøm, Geir Ringstad, Lars Magnus Valnes

**Affiliations:** 1 Institute of Clinical Medicine, Faculty of Medicine, University of Oslo, Oslo, Norway; 2 Department of Neurosurgery, Oslo University Hospital – Rikshospitalet, Oslo, Norway; 3 Oslo Centre of Biostatistics and Epidemiology, Research Support Services, Oslo University Hospital, Oslo, Norway; 4 Faculty of Health Sciences, Oslo Metropolitan University, Oslo, Norway; 5 Faculty of Medicine, University of Oslo, Oslo, Norway; 6 Department of Otorhinolaryngology, Surgical Division, Akershus University Hospital, Nordbyhagen, Norway; 7 Department of Radiology, Oslo University Hospital-Rikshospitalet, Oslo, Norway

**Keywords:** Sleep quality, brain metabolism, molecular clearance, magnetic resonance imaging, cerebrospinal fluid tracer

## Abstract

Chronic sleep disturbance is a risk factor for dementia disease, possibly due to impaired sleep-dependent clearance of toxic metabolic by-products. We compared enrichment of a cerebrospinal fluid (CSF) tracer within brain of patients reporting good or poor sleep quality, assessed by the Pittsburgh Sleep Quality Index (PSQI) questionnaire. Tracer enrichment in a selection of brain regions was assessed using multiphase magnetic resonance imaging up to 48 hours after intrathecal administration of the contrast agent gadobutrol (0.5 ml of 1 mmol/ml) serving as tracer. Tracer enrichment differed between patients with good (PSQI ≤5) and poor (PSQI >5) sleep quality in a cohort of non-dementia individuals (n = 44; age 42.3 ± 14.5 years), and in patients with the dementia subtype idiopathic normal pressure hydrocephalus (n = 24; age 71.0 ± 4.9 years). Sleep impairment was associated with increased CSF tracer enrichment in several brain regions. Cortical brain volume as well as entorhinal cortex thickness was reduced in the oldest cohort and was correlated with the severity of sleep disturbance and the degree of cortical tracer enrichment. We suggest chronic sleep disturbance is accompanied by altered glymphatic function along enlarged perivascular spaces.

## Introduction

Chronic sleep disturbance is a risk factor for dementia diseases and is commonly seen in dementias such as Alzheimer’s and Parkinson’s diseases,^1–4^ as well as in other dementing brain diseases, e.g. after traumatic brain injury (TBI).^5^ While it is disputed whether sleep disturbance is cause or consequence of dementia disease, impaired glymphatic function may represent a direct link between chronic sleep deprivation and the aggregation of toxic by-products of brain metabolism in dementia disease, i.e. Aβ and hyper-phosphorylated tau (HPτ) aggregation in Alzheimer’s disease and aggregation of α-synuclein in Parkinson’s disease.^6^ TBI may as well result in increased cerebral Aβ and HPτ burden and risk of Alzheimer’s.^7^ Currently, the sleep-dependent glymphatic concept is primarily based on studies of acute sleep interventions in rodents. In mice, sleep enhanced clearance of amyloid-β (Aβ) from cerebral cortex twofold due to a 60% increase of brain interstitial volume fraction.^8^ On the other hand, acute sleep deprivation in mice increased the interstitial level of Aβ and increased Aβ formation,^9^ as well as increased the amount of soluble amyloid-β and the risk of Aβ plaque formation,^10^ and increased the levels of HPτ in the interstitial fluid of the hippocampus.^11^ The findings in rodents have to some extent been translated to humans. A positron emission tomography (PET) study in cognitive healthy older adults gave evidence for an association between sleep quality and brain tau and amyloid-β burden, suggesting sleep quality to be a marker of early Alzheimer’s disease.^12^ In cognitively normal elderly people, slow wave sleep disturbance was associated with increased Aβ42 levels in CSF, indicating that disturbed sleep might increase soluble Aβ levels in the brain.^13^ One night of total sleep deprivation prevented the decrease in CSF Aβ42 levels seen after unrestricted sleep, and the authors hypothesized that chronic sleep disturbance increases Aβ42 levels.^14^ Furthermore, one night of sleep deprivation was in a human Aβ PET study found to increase parenchymal Aβ burden by 5%.^15^ Acute sleep deprivation in humans as well negatively affects cognitive functions such as memory, learning, attention and emotional reactivity.^16^ We recently reported that one night of total sleep deprivation reduced clearance of a cerebrospinal fluid (CSF) tracer from human brain.^17^ To which degree the results of acute sleep interventions relate to chronic sleep disturbance remains to be explored.

In this present study, we examined whether CSF tracer enrichment in human brain differs between individuals reporting subjective good or poor sleep quality on a general basis. A magnetic resonance imaging (MRI) contrast agent (gadobutrol; Bayer, GE) was applied as CSF tracer to enrich brain tissue after intrathecal injection.^18^ This CSF tracer is a hydrophilic molecule (molecular weight 604Da; hydraulic diameter about 2 nm) that does not pass the blood-brain-barrier (BBB), thereby distributing freely within the extra-vascular compartment of the brain.^18^ We hypothesize that the cerebral tracer passage is indicative of the extra-vascular transport of soluble by-products of cerebral metabolism such as Aβ, HPτ and α-synuclein, and that parenchymal tracer enrichment reflects glymphatic enhancement. To this end, we separately examined two cohorts of individuals, namely one cohort of individuals without any diagnosed dementia disease, and another cohort about three decades older, consisting of patients with the dementia subtype idiopathic normal pressure hydrocephalus (iNPH), which show histopathological similarities with Alzheimer’s disease.^19,20^

## Materials and methods

### Ethical permissions

The following authorities approved the study: The Regional Committee for Medical and Health Research Ethics (REK) of Health Region South-East, Norway (2015/96). The Institutional Review Board of Oslo university hospital (2015/1868). The National Medicines Agency (15/04932-7). The study was registered in Oslo University Hospital Research Registry (ePhorte 2015/1868), and conducted according to the ethical standards of the Helsinki Declaration (1975 and as revised in 1983). Study participants were included after written and oral informed consent. The study follows a prospective and observational design.

### Assessment of chronic sleep disturbance

Subjective sleep quality was assessed using the Pittsburgh Sleep Quality Index (PSQI) questionnaire, where patients were asked to describe their sleep over the past months, i.e. how their sleep is in general, not exclusively at the time of the MRI acquisitions. The PSQI was developed by Buysse et al.^21^ to measure sleep quality characteristics. We applied a Norwegian translation of the questionnaire.^22^ The PSQI questionnaire incorporates 25 questions wherein 19 questions are processed into a global score and seven domains: sleep duration, sleep disturbance, sleep onset latency, daytime dysfunction, habitual sleep efficiency, subjective sleep quality and use of sleeping medication.^21^ Each domain is scored from 0 to 3; the global scores have a range from 0 to 21, with higher scores indicative of poor sleep quality. The PSQI scores were dichotomized into good or poor sleep quality; a global PSQI score ≤5 is considered as indicative of good sleep quality.^23^ Notably, the notations good and poor sleepers refer to general sleep quality. The participants were instructed to report their subjective sleep quality over the last months, not referring to sleep quality over the last few days when MRI was performed.

### Study populations

The study population includes consecutive patients retrieved from a larger prospective research study of patients undergoing intrathecal contrast-enhanced MRI as part of their work-up of various CSF disorders within the Department of Neurosurgery at Oslo University Hospital, Norway. Intrathecal gadobutrol is currently given off-label on clinical indication; therefore, it is not used in healthy individuals. Among patients who had completed the PSQI questionnaire at the time of intrathecal contrast-enhanced MRI, this study included two patient cohorts that were defined according to the following criteria:

*Patient cohort #1 without dementia disease.* These patients underwent clinical work-up of tentative CSF disorders. The diagnosis categories with tentative CSF disturbances included patients with no verified CSF disturbance (also denoted reference subjects), or patients examined with MRI for symptoms related to arachnoid cysts or ventriculomegaly. First, all individuals within these categories who reported good sleep quality (i.e. global PSQI score ≤5) were identified. Then, for each individual with good sleep quality, the individuals with poor sleep quality matching closest in age and gender within the same diagnosis category were identified.

*Patient cohort #2 with the dementia subtype iNPH.* This patient cohort included individuals undergoing intrathecal contrast enhanced MRI as work-up for the dementia subtype iNPH. First, all iNPH patients reporting good sleep quality (i.e. global PSQI score ≤5) were identified. Subsequently, for each individual with good sleep quality, iNPH patients being closest in age and gender with poor sleep quality were identified.

The patient cohorts were defined prior to analysis of MRI and FreeSurfer data.

### Assessment of cerebral CSF tracer enrichment

To assess enrichment of a CSF tracer in human brain, the MRI contrast agent gadobutrol was used as CSF tracer. Intrathecal injection of gadobutrol in a dose of 0.5 mmol (0.5 ml of 1.0 mmol/ml gadobutrol; Gadovist, Bayer Pharma AG, Berlin, Germany) was performed by an interventional neuroradiologist. Standardized T1-weighted MRI scanning was performed before and at multiple time points after intrathecal gadobutrol: 0–0.5 hours, 0.5–2 hours, 2–4 hours, 4–7 hours (Day 1), 24 hours (Day 2), and 48 hours (Day 3).

Gadobutrol increases the T1 relaxation of water, which provides a higher T1 signal intensity at the image gray scale, providing a semi-quantitative measure of the tracer level. The images were post-processed using FreeSurfer software (version 6.0) to determine percentage change in normalized T1 signal units, indicative of tracer enrichment. Comparisons were done between individuals with good or poor sleep quality in both patient cohorts.

### MRI protocol and image analysis

The MRI protocol utilizes a 3 Tesla Philips Ingenia MRI scanner (Philips Medical systems, Best, The Netherlands), with equal imaging protocol settings at all time points to acquire sagittal 3D T1-weighted volume scans. The imaging parameters are as follows: repetition time = “shortest” (typically 5.1 ms), echo time = “shortest” (typically 2.3 ms), Flip angle = 8 degrees, field of view = 256 × 256 cm and matrix = 256 × 256 pixels (reconstructed 512 × 512). We sampled 184 over-contiguous (overlapping) slices with 1 mm thickness, which were automatically reconstructed to 368 slices with 0.5 mm thickness. The duration of each image acquisition was 6 minutes and 29 seconds. To secure consistency and reproducibility of the MRI slice placement and orientation, slice orientation of image stacks were defined using an automated anatomy recognition protocol based on landmark detection in MRI data (SmartExam™, Philips Medical Systems, Best, The Netherlands) for every time point.

FreeSurfer software (version 6.0) (http://surfer.nmr.mgh.harvard.edu/) was used for segmentation, parcellation and registration/alignment of the longitudinal data, and to determine the increase in T1 intensity caused by the CSF tracer, as previously reviewed.^24^ By means of a hybrid watershed/surface deformation procedure,^25^ non-brain tissue is removed, and segmentation of the subcortical white matter, deep gray matter structures (including hippocampus, amygdala, caudate, putamen and ventricles) is performed.^26,27^ We used the MR images of each patient to create a median template registered to the baseline;^28^ for each patient the MR images were registered to the corresponding template applying a rigid transformation.^28^ The registrations were checked manually by the senior author to correct for any registration errors.

We adjusted for changes in the gray-scale between MRI scans, by dividing the T1 signal unit for each time point by the T1 signal unit of a reference region of interest (ROI) for the respective time point. The reference ROI was placed within the posterior part of the orbit, as previously described.^17^ The ratio is the *normalized T1 signal unit*s, which corrects for baseline changes of image gray scale due to automatic image scaling.

For visualization, a median template image of each group was created for each time point, and a relative change in intensity from before intrathecal gadobutrol to 24 hours after gadobutrol was computed. Different template was chosen from each of the patient cohorts, and the image was constructed by taking the median value of each segmented region, and subsequent median over the cohort.

The FreeSurfer analysis also provided volume estimates of the regions of interest referred to in the study.

### MRI biomarkers of neurodegeneration

Here, we applied two MRI biomarkers of neurodegeneration.

#### Entorhinal cortex (ERC) thickness

The ERC thickness was measured in coronally reconstructed T1 volume acquisitions with 1 mm slice thickness at level of the hippocampal sulcus. ERC was measured from the ERC surface to the gray/white matter interface, and midway between the tentative location of parasubiculum and perirhinal cortex. The ERC thickness was previously found to be among the brain regions that best discriminate between cognitively normal subjects and patients with mild cognitive impairment or Alzheimer’s disease.^29^

#### Medial temporal atrophy (MTA)

We categorized the degree of MTA using the Scheltens score^30^ that is a visual rating of the width of the choroid fissure, the width of the temporal horn and the height of the hippocampal formation: Score 0 (no atrophy), score 1 (only widening of choroid fissure), score 2 (also widening of temporal horn of lateral ventricle), score 3 (moderate loss of hippocampal volume, decrease in height), and score 4 (severe volume loss of hippocampus).

### Statistical analyses

The statistical analysis was performed using SPSS version 26 (IBM Corporation, Armonk, NY) and Stata/SE 16.1 (StataCrop LLC, College Station, TX).

Continuous data were presented as mean (standard deviation) or mean (95% confidence intervals), as appropriate. We estimated from the image analysis the mean and standard error at 0 (pre-contrast), 0–0.5 hours, 0.5–2 hours, 2–4 hours, 4–7 hours, 24 hours, 48 hours, and at 4 weeks follow-up. Repeated measurements were examined with linear mixed models by maximum likelihood estimation using a subject-specific random intercept. Using estimated marginal mean from the statistical model, we tested the difference between the individuals with good or poor sleep quality at the different points. Moreover, we applied multivariable analysis to calculate the impact of differences in gender and age between groups and examine impact of individual PSQI subcategories on tracer enhancement after 4–7 and 24 hours. The distribution of the assumed normal distributed random effects and residuals from the statistical model was assessed using both the Shapiro–Wilk normality test and descriptive statistics with boxplots and histograms. It was also conducted for other data in both groups. Correlations between different variables were examined using Pearson correlation test.

Statistical significance was accepted at the .05 level (two-tailed).

### Data availability

The data presented in this work is available upon reasonable request.

## Results

### Patient populations

Patient cohort #1 without diagnosed dementia disease included 44 individuals who were examined for tentative CSF disturbances, such as symptomatic arachnoid cysts and ventriculomegaly. The average age of these individuals was 42.3 ± 14.5 years, with no differences in age or gender between good (n = 17) or poor (n = 27) sleepers (Table 1). The PSQI global score was 3.4 ± 1.0 for good sleepers and 11.2 ± 2.9 for poor sleepers, with differences in the PSQI subcategories between good/poor sleepers (Table 1).

**Table 1.** Demographic and clinical information about the two study groups.

	Cohort #1 (non-dementia subjects)			Cohort #2 (Dementia subtype iNPH)		
	Good sleepers	Poor sleepers	Significance	Good sleepers	Poor sleepers	Significance
*N*	17	27		12	12	
Sex (F/M)	10/7	19/8	ns	2/10	4/8	ns
Age (years)	42.7 ± 15.1	42.0 ± 14.3	ns	70.7 ± 5.4	71.3 ± 4.7	ns
BMI (kg/m^2^)	27.7 ± 3.7	27.1 ± 5.4	ns	28.2 ± 4.5	26.9 ± 4.4	ns
Cognitive measures	–	–				
iNPH subscore^1^	–	–		4.2 ± 0.4	3.9 ± 0.3	ns
MMSE^2^	–	–		23.0 ± 4.3	25.9 ± 2.5	ns
Sleep quality						
PSQI Global score	3.35 ± 1.00	11.15 ± 2.85	P < 0.001	3.75 ± 1.29	8.33 ± 2.19	P < 0.001
PSQI Sub-categories						
Sleep duration	0.18 ± 0.39	1.33 ± 1.14	P < 0.001	0.42 ± 0.90	0.83 ± 0.84	ns
Sleep disturbance	1.00 ± 0.00	1.04 ± 0.19	ns	1.00 ± 0.00	1.08 ± 0.29	ns
Sleep onset latency	0.41 ± 0.51	2.11 ± 0.97	P < 0.001	0.42 ± 0.52	1.67 ± 1.08	P = 0.001
Daytime dysfunction	0.88 ± 0.33	1.26 ± 0.53	P = 0.012	1.00 ± 0.43	1.00 ± 0.43	ns
Habitual sleep efficiency	0.18 ± 0.53	1.81 ± 1.08	P < 0.001	0.25 ± 0.45	1.67 ± 0.98	P < 0.001
Subjective sleep quality	0.71 ± 0.59	2.11 ± 0.75	P < 0.001	0.42 ± 0.52	1.25 ± 0.45	P < 0.001
Use of sleep medication	0.00 ± 0.00	1.48 ± 1.31	P < 0.001	0.25 ± 0.62	0.83 ± 1.34	ns
Biomarkers of neurodegeneration						
ERC thickness (mm)	2.41 ± 0.23	2.33 ± 0.27	ns	2.16 ± 0.19	1.74 ± 0.30	P < 0.001
Grade 0	5 (29%)	11 (41%)		0	0	
Grade 1	11 (65%)	13 (48%)		0	1 (8%)	
Scheltens MTA						
Grade 2	1 (6%)	3 (11%)	ns	9 (75%)	8 (67%)	ns
Grade 3	0	0		3 (25%)	3 (25%)	
Brain volume measures						
Volume Cerebral cortex (ml)	500 ± 45	504 ± 43	ns	497 ± 37	464 ± 37	P = 0.019
Volume frontal cortex (ml)	197 ± 22	200 ± 19	ns	197 ± 17	184 ± 17	P = 0.031
Volume temporal cortex (ml)	108 ± 11	107 ± 10	ns	104 ± 9	97 ± 6	P = 0.020
Volume parietal cortex (ml)	140 ± 12	140 ± 14	ns	140 ± 13	130 ± 12	P = 0.037
Volume occipital cortex (ml)	55 ± 6	56 ± 7	ns	56 ± 4	53 ± 7	ns
Volume 4th ventricle (ml)	2.1 ± 1.2	1.7 ± 0.8	ns	3.2 ± 1.3	3.4 ± 1.9	ns
Volume 3rd ventricles (ml)	1.6 ± 0.9	1.2 ± 0.7	ns	3.7 ± 0.6	3.8 ± 1.7	ns
Volume lateral ventricles (ml)	36 ± 25	27 ± 21	ns	145 ± 33	155 ± 66	ns

Categorical data presented as numbers; continuous data presented as mean ± standard deviation. Significant differences between groups were determined by independent samples t-test for continuous data and by Pearson Chi-square test for categorical data. Ns: non-significant. The subjective sleep quality from Day 1 to Day 2 is indicated. Cortical brain volume measures refer to gray matter.

The patient cohort #2 examined for iNPH included 24 patients who fulfilled the diagnosis probable iNPH according to the American-European guidelines.^31^ The average age was 71.0 ± 4.9 years. The good (n = 12) and poor (n = 12) sleepers did not differ with regard to age and gender (Table 1). Good sleepers had a global PSQI score of 3.8 ± 1.29 and the poor sleepers a global PSQI score of 8.6 ± 2.63 (Table 1). PSQI sub-categories differed between groups (Table 1).

With regard to MRI biomarkers of neurodegeneration, the ERC was thicker in patient cohort #1 without dementia disease than in the cohort #2 with the dementia subtype iNPH (2.36 ± 0.26 mm vs. 1.95 ± 0.33 mm; P < 0.001; independent samples t-test). Furthermore, the distribution of MTA score differed significantly between cohorts #1 and #2 [Cohort #1: MTA 0 (36%), MTA 1 (55%), MTA 2 (9%), MTA 3 (0%); Cohort #2: MTA 0 (0%), MTA 1 (4%), MTA 2 (71%), MTA 3 (25%). P < 0.001; Pearson chi-square test].

While ERC thickness was not significantly different between good and poor sleepers of cohort #1, the ERC thickness was significantly thinner among poor sleepers of cohort #2 (Table 1).

There were no differences between good and poor sleepers of cohort #1 with regard to volumes of cerebral cortex, regional volumes of gray matter in frontal, temporal, parietal or occipital cortex, or ventricular volume measures (Table 1). On the other hand, poor sleepers of cohort #2 presented with significantly reduced volume of cerebral cortex, including gray matter of frontal, temporal and parietal cortex, but ventricular volume measures were comparable between groups (Table 1).

### CSF tracer enrichment within CSF of individuals without or with dementia disease (cohorts #1 and #2)

The enrichment of tracer within CSF was similar over time in good and poor sleepers of cohort #1 (Supplementary Figure 1). On the contrary, in cohort #2 with the dementia disease iNPH, tracer enrichment within CSF was significantly increased at 4–7 hours (mean difference 411%; P < 0.001; Supplementary Figure 2). This latter finding indicates reduced CSF turnover in iNPH subjects.

### CSF tracer enrichment in brain of individuals without dementia disease (cohort #1)

**Figure 1.**
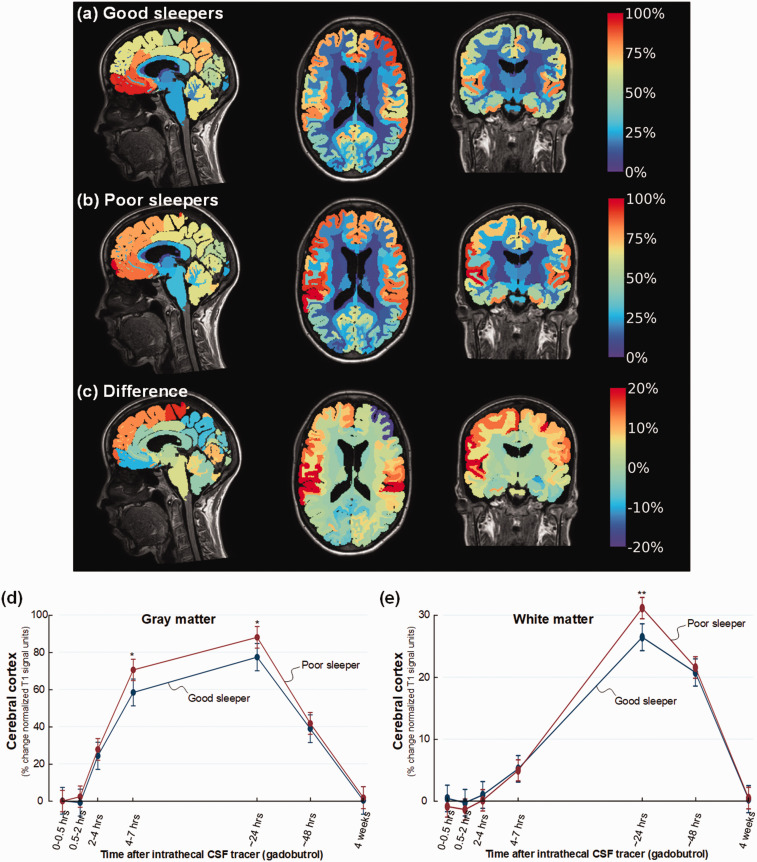
Color maps illustrating levels of CSF tracer enrichment in non-dementia individuals having good or poor sleep quality. For patient cohort #1 with no diagnosed dementia disease, CSF tracer enrichment in brain tissue is given as percentage increase of normalized T1 MRI signal units from baseline until 24 hours, and presented at a color scale. Tracer in CSF spaces has been subtracted. (a) The average increase in normalized cerebral T1 signal from baseline until 24 hours for the individuals with good sleep quality (PSQI score ≤5) is presented as mid-sagittal (left), mid-axial (middle), and mid-coronal (right) slices (n = 17; Good sleepers). (b) The average percentage normalized T1 signal increase from baseline until 24 hours is shown for the individuals with poor sleep quality (PSQI score >6) (n = 27; Poor sleepers). (c) The difference in percentage normalized T1 signal increase between Poor sleepers and Good sleepers is shown. The color maps demonstrate higher tracer levels in Poor sleepers with red color indicating the highest tracer levels, which we interpret as glymphatic enhancement. Blue color and negative values are indicative of accelerated clearance of tracer. (d) Trend plots of percentage change in normalized T1 signal unit within gray matter of cerebral cortex demonstrating significant differences between the Poor sleepers (red line) and Good sleepers (blue line) at 4–7 and 24 hours. (e) Trend plots of percentage change in normalized T1 signal units within white matter demonstrating significant differences between the Poor sleepers (red line) and Good sleepers (blue line) at 24 hours.*P < 0.05, **P < 0.01, ***P < 0.001. Trend plots show mean with error bars (95% confidence intervals) from linear mixed models.

Figure 1(a) to (c) illustrates the stronger enrichment of CSF tracer within brain of poor sleepers in cohort #1 at 24 hours. Poor sleepers demonstrated increased tracer enrichment in cerebral cortex at 4–7 hours (mean difference 12%, P = 0.010) and 24 hours (mean difference 11%, P = 0.026) (Figure 1(d)), and in subcortical white matter at 24 hours (difference 5%, P = 0.001) (Figure 1(e)).

**Figure 2.**
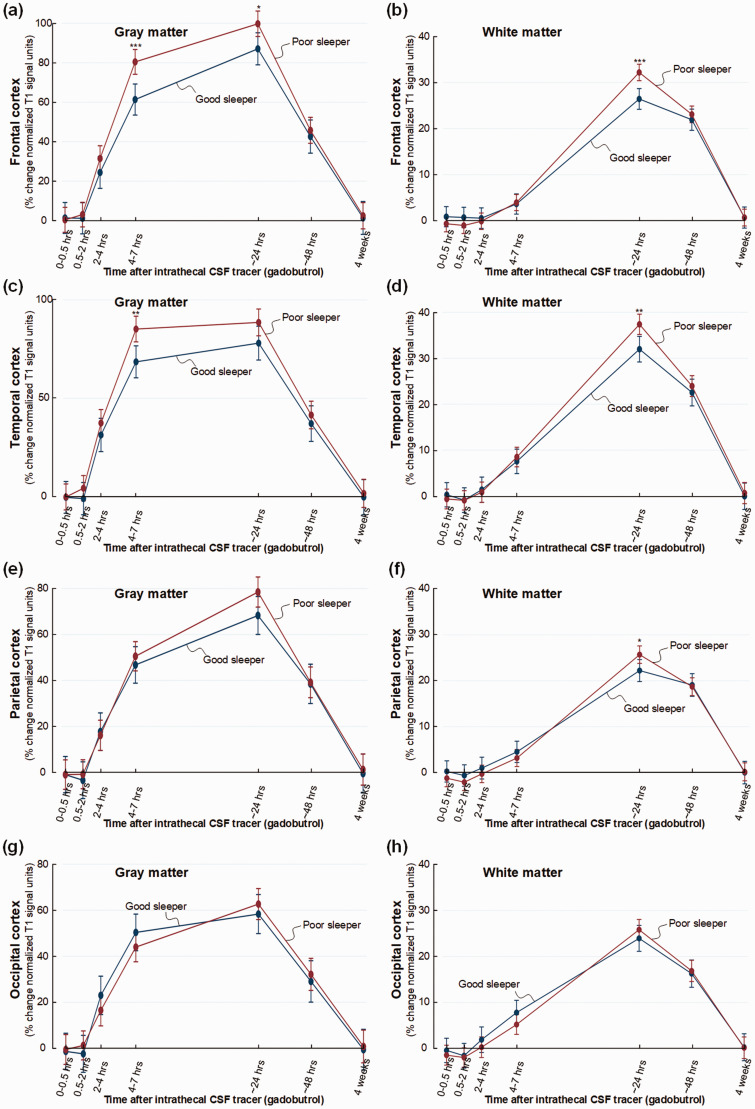
Trend plots demonstrating differences in CSF tracer enrichment in the four brain lobes of non-dementia subjects with good or poor sleep quality. For patient cohort #1 with no diagnosed dementia disease, trend plots of percentage change in normalized T1 signal units from baseline, indicative of glymphatic enhancement, are presented for (a) gray matter of frontal cortex, (b) subcortical white matter within frontal cortex, (c) gray matter of temporal cortex, (d) white matter within temporal cortex, (e) gray matter of parietal cortex, (f) white matter within parietal cortex, (g) gray matter of occipital cortex, and (h) white matter within occipital cortex. Significant differences between the Poor sleepers (red line) and Good sleepers (blue line) are indicated as follows: *P < 0.05, **P < 0.01, ***P < 0.001. Trend plots show mean with error bars (95% confidence intervals) from linear mixed models.

There was some regional variation in tracer enrichment. In frontal cortex, tracer enrichment was increased in poor sleepers in gray matter after 4–7 (mean difference 19%, P < 0.001) and 24 hours (mean difference 13%, P = 0.017) and in white matter after 24 hours (mean difference 6%, P < 0.001) (Figure 2(a) and (b)). Tracer enrichment was significantly increased in temporal cortex gray matter at 4–7 hours (mean difference 17%, P = 0.002) and in white matter after 24 hours (mean difference 5%, P = 0.003) (Figure 2(c) and (d)). The parietal cortex revealed no change in enrichment in gray matter, but in white matter after 24 hours (mean difference 4%, P = 0.026) (Figure 2(e) and (f)). No significant differences in tracer enrichment were seen in the occipital gray and white matter (Figure 2(g) and (h)).

**Figure 3.**
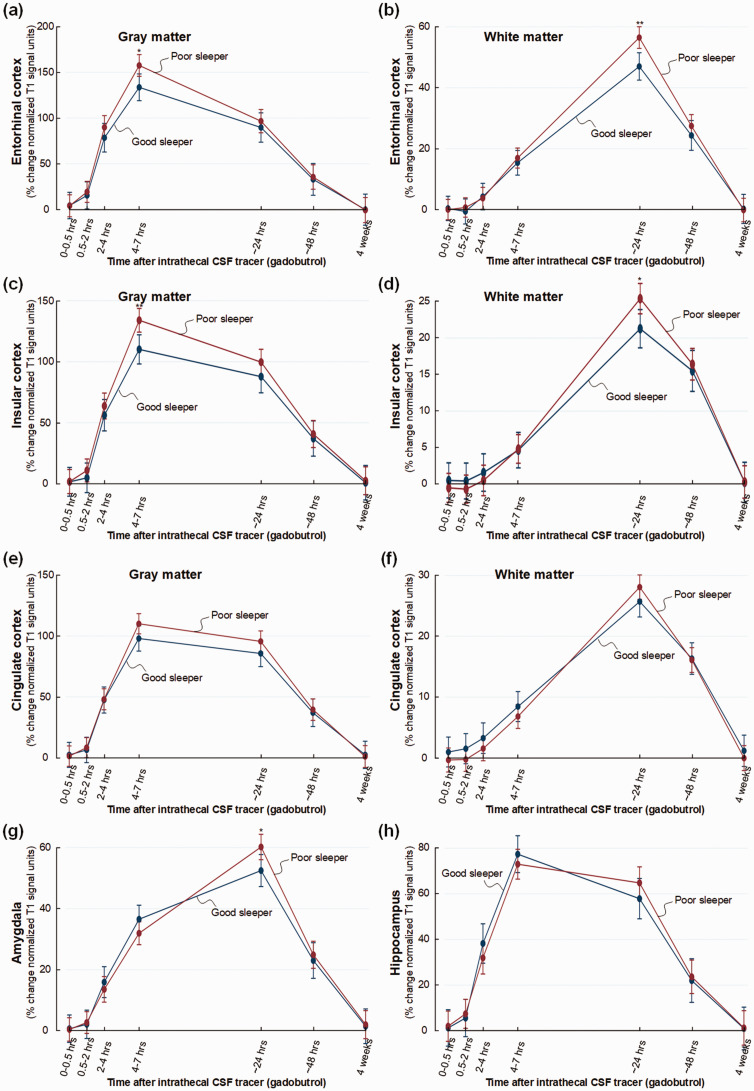
Trend plots illustrating CSF tracer enrichment in selected brain regions of non-dementia subjects with good or poor sleep quality. For patient cohort #1 with no diagnosed dementia disease, trend plots of percentage change in normalized T1 signal units from baseline, indicative of extra-vascular cerebral tracer enrichment in (a) gray matter of entorhinal cortex, (b) white matter within entorhinal cortex, (c) gray matter of insular cortex, (d) white matter within insular cortex, (e) gray matter of cingulate cortex, (f) white matter within cingulate cortex, (g) amygdala and (h) hippocampus. Significant differences between the Poor sleepers (red line) and Good sleepers (blue line) are indicated as follows: *P < 0.05, **P < 0.01, ***P < 0.001. Trend plots show mean with error bars (95% confidence intervals) from linear mixed models.

Some other regions should be noted. Poor sleeper showed increased tracer enrichment in entorhinal cortex at 4–7 hours (mean difference 24%, P = 0.013) and in white matter at 24 hours (mean difference 10%, P = 0.001) (Figure 3(a) and (b)). Similarly, poor sleepers presented significantly stronger tracer enrichment in insular cortex after 4–7 hours (mean difference 24%, P = 0.002) and after 24 hours in subinsular white matter (mean difference 4%, P = 0.016) (Figure 3(c) and (d)). Tracer enrichment was not significantly changed in cingulate cortex of poor sleepers (Figure 3(e) and (f)), but was significantly increased after 24 hours in amygdala of poor sleepers (mean difference 8%, P = 0.024) (Figure 3(g)). Poor sleepers showed no change in tracer enrichment in hippocampus (Figure 3(h)).

With regard to the PSQI subcategories, we found no impact on tracer enrichment in cerebral cortex gray matter after 4–7 or 24 hours (data not shown).

### CSF tracer enrichment in brain of individuals with the dementia subtype iNPH (cohort #2)

**Figure 4.**
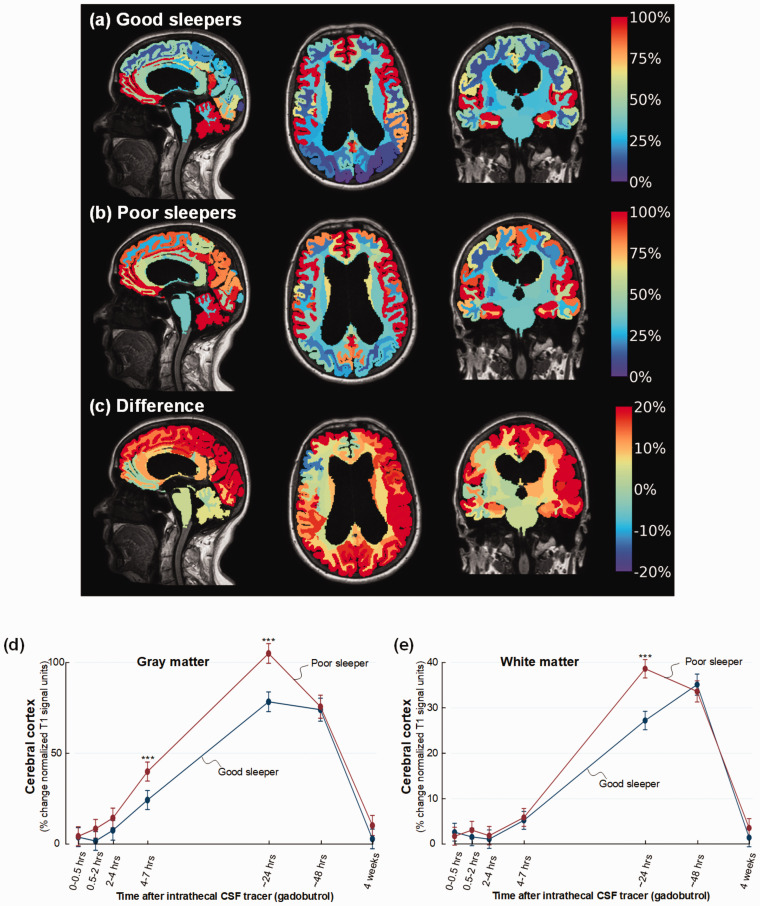
Increased CSF tracer enrichment in iNPH patients with poor sleep quality. For patient cohort #2 with the dementia subtype iNPH, CSF tracer enrichment in brain tissue is presented as percentage increase of normalized T1 MRI signal units from baseline until 24 hours, and indicated at the color scale. Tracer in CSF spaces has been subtracted. (a) The average increase in cerebral normalized T1 signal units from baseline until 24 hours for the iNPH cohort with good sleep quality is presented as sagittal (left), axial (middle), and coronal (right) MRI scans, with percentage change shown at the color scale (n = 12; Good sleepers). (b) The average percentage increase in normalized T1 signal units from baseline until 24 hours is shown for the cohort with poor sleep quality (n = 12; Poor sleepers). (c) The difference in percentage increase in normalized T1 signal units between the Poor sleepers and the Good sleepers is shown. The color maps demonstrate stronger tracer enrichment in Poor sleepers with red color indicating the highest tracer levels, which is interpreted as glymphatic enhancement. Blue color and negative values are indicative of accelerated clearance of tracer. (d) Trend plots of percentage change in normalized T1 signal units within cerebral cortex of iNPH subjects demonstrating significant differences between the Poor sleepers (red line) and Good sleepers (blue line) at 4-7 and 24 hours. (e) Trend plots of percentage change in normalized T1 signal units within white matter of cerebral cortex demonstrating significant differences between the Poor sleepers (red line) and Good sleepers (blue line) at 24 hours.*P < 0.05, **P < 0.01, ***P < 0.001. Trend plots show mean with error bars (95% confidence intervals) from linear mixed models.

Figure 4(a) to (c) illustrates the stronger cerebral enrichment of CSF tracer that characterizes poor sleepers of cohort #2 at 24 hours. Among poor sleepers, the enrichment of CSF tracer in cerebral cortex was significantly stronger at 4–7 hours (mean difference 16%, P < 0.001) and after 24 hours (mean difference 26%, P < 0.001) (Figure 4(d)), and significantly increased enrichment in white matter at 24 hours (mean difference 11%, P < 0.001) (Figure 4(e)).

**Figure 5.**
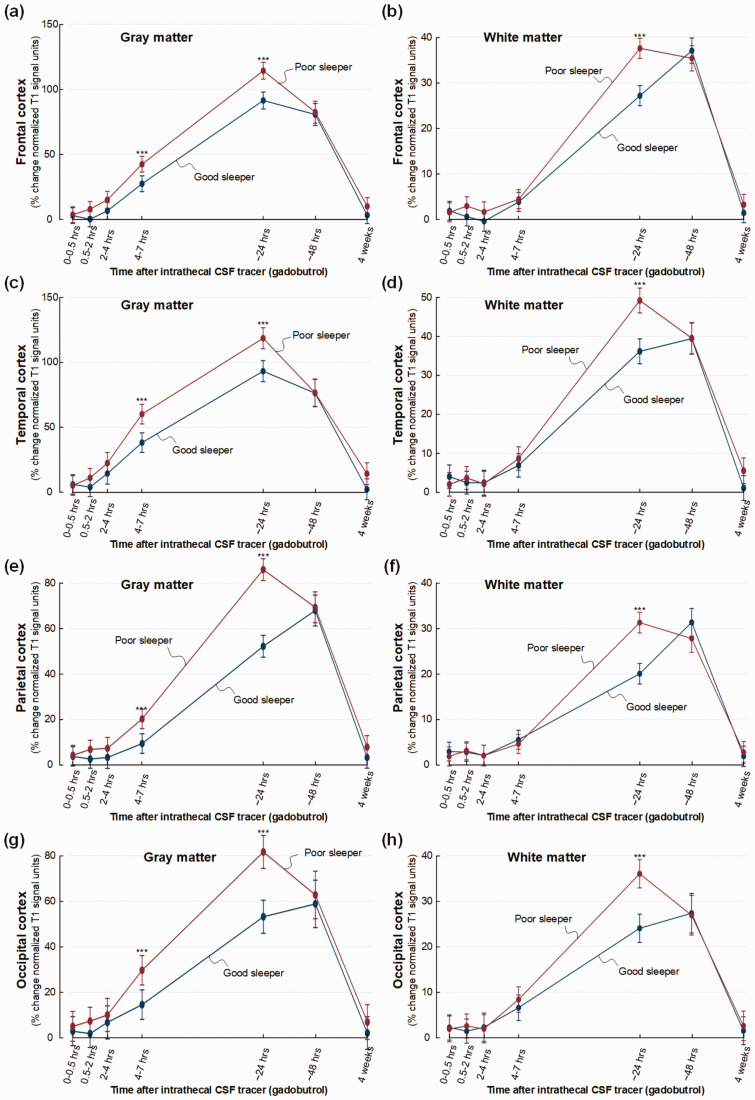
Trend plots illustrating CSF tracer enrichment in the four brain lobes of iNPH patients with good or poor sleep quality. For patient cohort #2 with the dementia subtype iNPH, trend plots of percentage change in normalized T1 signal units from baseline, indicative of extra-vascular cerebral tracer enrichment are presented for (a) gray matter of frontal cortex, (b) white matter of frontal cortex, (c) gray matter of temporal cortex, (d) white matter within temporal cortex, (e) gray matter of parietal cortex, (f) white matter within parietal cortex, (g) gray matter of occipital cortex, and (h) white matter within occipital cortex. Significant differences between the Poor sleepers (red line) and Good sleepers (blue line) are indicated as follows: *P < 0.05, **P < 0.01, ***P < 0.001. Trend plots show mean with error bars (95% confidence intervals) from linear mixed models.

The increase in cortical tracer enrichment in poor sleepers showed a similar pattern in all lobes of the brain. Hence, cortical tracer enrichment was significantly increased in poor sleepers in frontal gray matter at 4–7 hours (mean difference 15%, P < 0.001) and 24 hours (mean difference 23%, P < 0.001) and in white matter at 24 hours (mean difference 10%, P < 0.001) (Figure 5(a) and (b)), in temporal gray matter at 4–7 hours (mean difference 22%, P < 0.001) and 24 hours (mean difference 25%, P < 0.001) and white matter at 24 hours (mean difference 13%, P < 0.001) (Figure 5(c) and (d)), in parietal gray matter at 4–7 hours (mean difference 11%, P < 0.001) and 24 hours (mean difference 34%, P < 0.001) and parietal white matter at 24 hours (mean difference 11%, P < 0.001) (Figure 5(e) and (f)), as well as in occipital gray matter at 4–7 hours (mean difference 15%, P < 0.001) and 24 hours (mean difference 29%, P < 0.001) and white matter at 24 hours (mean difference 12%, P < 0.001) (Figure 5(g) and (h)).

**Figure 6.**
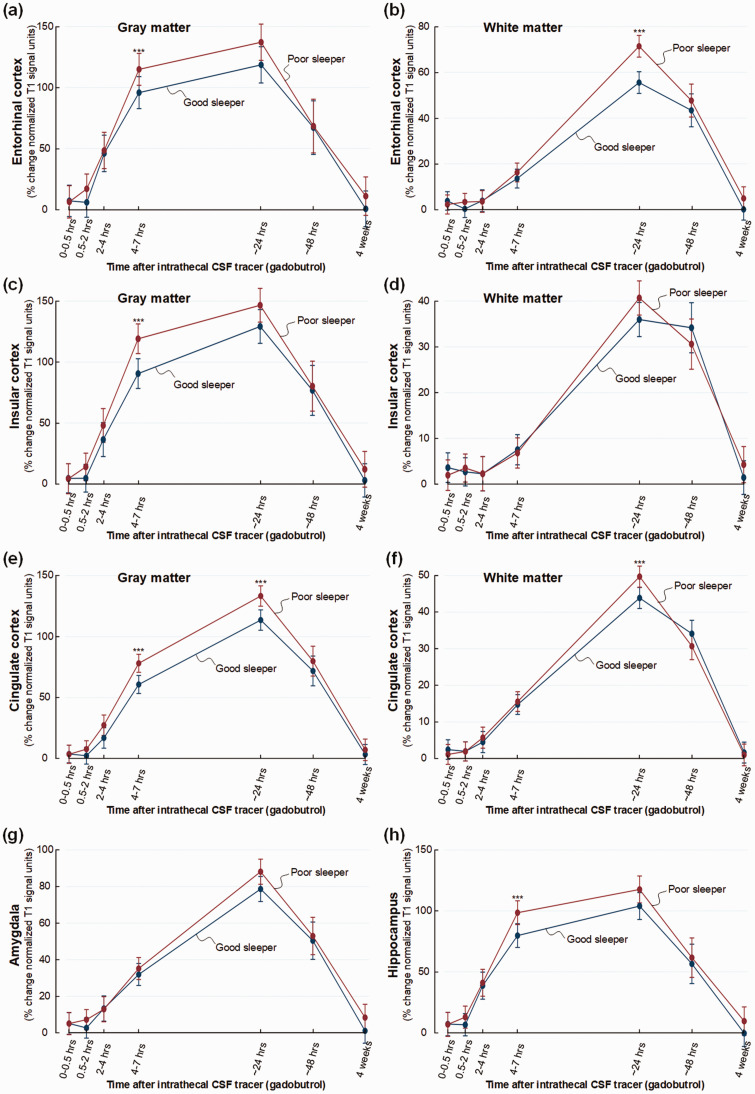
Increased CSF tracer enrichment in selected brain regions of iNPH patients of cohort #2 with good or poor sleep quality. For patient cohort #2 with the dementia subtype iNPH, trend plots of percentage change in normalized T1 signal units from baseline, indicative of extra-vascular cerebral tracer enrichment are presented for (a) gray matter of entorhinal cortex, (b) white matter of entorhinal cortex, (c) gray matter of insular cortex, (d) white matter of insular cortex, (e) gray matter of cingulate cortex, (f) white matter of cingulate cortex, (g) amygdala and (h) hippocampus. Significant differences between the Poor sleepers (red line) and Good sleepers (blue line) are indicated as follows: *P < 0.05, **P < 0.01, ***P < 0.001. Trend plots show mean with error bars (95% confidence intervals) from linear mixed models.

Poor sleepers in the cohort #2 of iNPH patients, showed increased tracer enrichment in entorhinal cortex after 4–7 hours (mean difference 19%, P < 0.001) and in white matter of entorhinal cortex after 24 hours (mean difference 16%, P < 0.001) (Figure 6(a) and (b)), increased tracer enrichment in insular cortex at 4–7 hours (mean difference 29%, P < 0.001) but not in subinsular white matter (Figure 6(c) and (d)). Moreover, poor sleepers showed increased tracer enrichment in cingulate cortex at 4–7 hours (mean difference 17%, P < 0.001) and 24 hours (mean difference 20%, P < 0.001), and cingulate subcortical white matter at 24 hours (mean difference 6%, P < 0.001) (Figure 6(e) and (f)). Tracer enrichment was not increased in amygdala of poor sleepers (Figure 6(g)), but was increased in hippocampus of poor sleepers after 4–7 hours (mean difference 19%, P < 0.001) (Figure 6(h)).

Concerning the PSQI subcategories, subjective sleep quality had a significant effect on tracer enrichment in cerebral cortex gray matter after 4–7 hours (P = 0.034) and daytime dysfunction had a significant effect on tracer enrichment in cerebral cortex gray matter after 24 hours (P = 0.039).

### Correlations between chronic sleep quality, brain volume and CSF tracer enhancement (cohorts #1and #2)

In cohort #1, we found no significant correlations between PSQI global score, cerebral cortex volume, entorhinal cortex thickness or tracer enrichment within cerebral cortex after 4–7 or 24 hours (data not shown).

In cohort #2, increasing PSQI global score, indicative of worse sleep quality, was associated with non-significant reduction in cerebral cortex volume (R = −0.39, P = 0.063), but significant reduction of entorhinal cortex thickness (R = −0.45, P = 0.027) (Supplementary Figure 3). With regard to the PSQI sub-categories, there was a significant negative correlation between cerebral cortex volume and sleep onset latency (R = −0.53, P = 0.007), and between entorhinal cortex thickness and both sleep onset latency (R = −0.59, P = 0.002) and subjective sleep quality (R = −0.42, P = 0.042) (data not shown).

Concerning tracer enrichment in individuals with the dementia subtype iNPH (cohort #2), there was a significant positive correlation between PSQI global score and tracer enrichment in cerebral cortex after 4–7 hours (R = 0.48, P = 0.017), but the correlation between PSQI global score and tracer enrichment in cerebral cortex after 24 hours was non-significant (R = 0.38, P = 0.070) (Supplementary Figure 4). Moreover, increasing tracer enrichment in cerebral cortex after 4–7 hours was associated with reduced cerebral cortex volume (R = −0.54, P = 0.006), while the association between tracer enrichment in cerebral cortex after 24 hours and volume of cerebral cortex was non-significant (R = −0.37, P = 0.074) (Supplementary Figure 5). We found no association between volumes of 4th, 3rd or lateral ventricles and either PSQI global score or cortical tracer enrichment (data not shown).

## Discussion

The present study shows increased CSF tracer enrichment within brain of humans with subjective poor sleep quality. Findings among poor sleepers were to a large extent comparable between a younger, non-dementia patient cohort (#1), and patients about three decades older with a dementia subtype (#2). Poor sleepers of the latter group had reduced volume of cerebral cortex and the increased tracer enrichment was accompanied with volume reduction of the cerebral cortex.

In both patient cohorts #1 and #2, tracer enrichment in poor sleepers was significantly increased in several gray matter locations both at 4–7 and 24 hours, and in white matter locations at 24 hours. Increased perivascular space volume in white matter has previously been associated with poor sleep quality,^32^ however, perivascular spaces within cortex cannot be visualized directly at MRI due to their small size, and their presence have even remained controversial as CSF influx routes.^33^ Nevertheless, it has later been shown that perfusion-fixation of mouse brain causes a 10-fold reduction in perivascular space size/dimension,^34^ and that perivascular spaces indeed represent low resistance pathways for CSF flow/influx to brain.^35^ Our present in vivo observations of increased cerebral CSF tracer enrichment in subjects with impaired sleep can therefore probably be attributed to increased perivascular space size and further speak in favor of perivascular spaces to be a main factor for CSF influx to human cortex, and not diffusion alone. To this end, increased load of enlarged perivascular spaces have been found in Alzheimer’s disease and mixed dementia compared to cognitively normal subjects, and enlarged perivascular space burden is also associated with tau and amyloid-B pathology.^36^ Sleep deprivation increases interstitial space fluid and CSF tau as well as tau pathology spreading,^11^ while tau aggregation may have a role in the bidirectional relationship between sleep and Alzheimer’s disease.^37^

Furthermore, in dementia patients having poor sleep, increased cerebral tracer levels at 4–7 and 24 hours possibly relate to lower clearance rate from CSF and thus higher levels of tracer within the CSF space of these individuals (Supplementary Figure 2). We have previously shown that the availability of tracer within CSF is positively correlated with the degree of tracer enrichment within the brain.^18,38^ This observation of impaired CSF turnover in poor sleepers may indicate that chronic sleep impairment acts differently on CSF efflux pathways than acute and total sleep deprivation, where it was recently shown that one night of sleep deprivation did not affect clearance from CSF in humans.^39^

It is of note that despite about three decades of age differences between patient cohorts #1 and #2, observations on cerebral tracer enrichment were to a large extent comparable. This strengthens the validity of our observations. Since the presently used CSF tracer is used off-label and given intrathecal on clinical indication, healthy individuals could not be included. Patient cohort #1 included individuals examined for tentative CSF disturbances, though none had a dementia diagnosis. This group was on average about three decades younger than patient cohort #2, which included individuals diagnosed with probable iNPH according to the European-American guidelines.^31^ The iNPH disease is a dementia subtype showing histopathological similarities with Alzheimer’s disease as a significant proportion of iNPH patients present with HPτ and Aβ deposition within the brain and with a definite risk of evolving from iNPH to Alzheimer’s disease.^20,40–45^ In several ways, iNPH may thus be considered a model of Alzheimer’s disease.^19^

An interesting observation was that poor sleepers of the iNPH cohort presented with reduced cerebral cortex volume and reduced volumes of the frontal, temporal and parietal lobes. Cerebral cortex volume reduction was as well accompanied with increasing PSQI global score, i.e. worse sleep. Chronic sleep disturbance may be associated with increased cortical atrophy,^46^ and volume loss such as in hippocampus and posterior cingulate cortex.^47^ In the present study, reduced cerebral cortex volume was associated with increased CSF tracer enrichment in cerebral cortex, indicating that increased perivascular space dimensions in the cortex is associated with cortical atrophy.

The differences between the patient cohorts were further illustrated by reduced ERC thickness and higher MTA score in cohort #2 with the dementia subtype, as compared with cohort #1 without dementia disease. It has previously been shown that cognitive decline and early dementia associates with higher MTA-score^30^ as well as thinning or volume loss of the ERC.^48–53^ Moreover, ERC thinning was accompanied with increased postmortem neurofibrillary tangle burden and increased Aβ load;^49^ early Alzheimer’s disease demonstrated profound degeneration of ERC layer II.^54^ The ERC-hippocampus circuit plays a fundamental role for cognitive function, such as memories for locations and events.^55–58^ It may therefore be of particular significance that the ERC thickness was significantly reduced among poor sleepers in the present iNPH cohort. We have previously shown reduced clearance of CSF tracer from ERC in subjects with iNPH compared to reference subjects, and proposed that reduced clearance of toxic waste products from ERC may be a mechanism behind the neurodegeneration and thinning of ERC seen in iNPH.^59^ The present findings extend our previous observations by showing impaired clearance of tracer from ERC in poor sleepers of both patient cohorts #1 and #2.

We here characterized chronic sleep disturbance utilizing the PSQI questionnaire that was developed by Buysse et al. in 1988 for people to self-score sleep quality.^21^ The PSQI questionnaire has since been a validated instrument for measuring subjective sleep quality.^22,23^ A global PSQI score >5 was previously found to indicate poor sleep quality with a diagnostic sensitivity of about 90% and specificity of 87%.^21^ As shown in Table 1, the global PSQI score differed substantially between good and poor sleepers in both cohorts #1 and #2.

We suggest the present data provide additional evidence for a sleep-dependent glymphatic system in humans. The glymphatic system was first described in rodent brain,^60^ and constitutes a paravascular pathway for convective transport of fluids and solutes along the arterial brain vessels, via interstitial tissue and with efflux along the venous brain vessels.^60^ The rodent glymphatic system seems to be primarily active during sleep.^8^ Our human in vivo findings supporting the glymphatic concept relies on intrathecal contrast-enhanced MRI studies: (1) Consistent observations of antegrade transport of CSF tracer along arteries.^38^ (2) Evidence for transport faster than extra-cellular diffusion in the observed tracer movement.^61^ (3) Evidence of sleep-dependent tracer enrichment within brain tissue.^17^ (4) Centripetal enrichment of brain tissue from outside cerebral cortex of a tracer strictly confined outside vessels due to the blood-brain-barrier.^18^ Concerning the latter, it should be noticed that the tracer enriches the brain centripetally while soluble waste is expected to be cleared centrifugally under physiological conditions.^60,62^ Therefore, different from clearance of endogenous byproducts of brain metabolism, the presently used CSF tracer is cleared from brain to CSF against a concentration gradient, as there is continuously presence of an assumedly higher concentration of tracer in the subarachnoid space through 48 hours, at least to some extent. Clearance failure caused by poor sleep quality may thus be underestimated by the presently used method.

A crucial question is whether the altered tracer movement in poor sleepers is cause or consequence of sleep disturbance. Most likely, several mechanisms are at play. In dementia, sleep disturbance could be caused by loss of neurons that are instrumental for sleep function such as neurons of the hypothalamic suprachiasmatic nucleus^63^ that senses light via the retinohypothalamic tract and regulates circadian rhythm.^64,65^ Moreover, Alzheimer’s disease is accompanied with degeneration of locus coeruleus that cause increased noradrenergic tone.^66–68^ The latter might affect paravascular molecular transport. In mice, increased noradrenergic activity, which is characteristic for the wake state, was accompanied with reduced glymphatic function.^8^ These authors proposed that the locus coeruleus adrenergic-mediated activity during sleep causes shrinkage of cell volume, increased interstitial space, and reduced resistance to diffusion or convective flow, which enhance clearances of waste solutes (e.g. amyloid-β) from brain parenchyma. In line with this reasoning, the authors reported that cortical interstitial volume fraction was 13-15% during the awake state while 22-24% in the sleeping or anesthetized state.^8^ Accordingly, impaired clearance of toxic waste may be suggested to lead to a vicious cycle wherein a resulting neurodegeneration in turn causes damage to brain areas involved in sleep-wake state, as well as locus coerulus adrenergic-regulated paravascular molecular transport.

Some limitations with the study should be noted. We matched the good and poor sleepers in each cohort best possible regarding age and gender, but some differences were noted even though they were non-significant. On the other hand, multivariable analysis considering the age and gender differences did not affect the results of statistical analysis. The PSQI data presented here refer to subjective sleep quality in general, but were not able to define the duration of poor sleep quality. Neither were objective sleep measures recorded, nor sleep quality during the 48 hours image acquisition period in particular. These aspects could be included in future studies. Moreover, we here included patients who underwent MRI on clinical indication for tentative CSF disturbance, which by itself might affect sleep function. Therefore, we cannot decipher enrichment of tracer in healthy brain from these data. Finally, to which degree our CSF tracer estimates clearance of metabolic waste products such as Aβ, HPτ and α-synuclein needs to be further explored. Another aspect worth mentioning is that the time course profiles of tracer enrichment within brain differs between this study and our previous sleep deprivation study.^17^ The reason for this is that patients with different underlying diseases were included in the two studies. Our former sleep deprivation study^17^ included patients with other underlying diseases such as 10/24 patients with idiopathic intracranial hypertension. This latter patient group presents with a faster clearance of tracer from brain.^69^ In comparison, tracer enhancement is more protracted in iNPH.^18^ How poor sleep quality affects tracer enrichment in human brain depending on underlying disease needs to be further explored.

In conclusion, the present study provides in vivo evidence for increased CSF tracer enrichment in brain of individuals with poor sleep quality, indicative of glymphatic enhancement. Comparable findings were made in two patient cohorts without or with dementia disease, and separated in age by about three decades. Increased CSF tracer influx in brain associated with chronic sleep impairment is suggestive of enlarged perivascular spaces in cortex, which was previously observed in patients with dementia and tau pathology. Altered glymphatic function may be accompanied with neurodegeneration. To this end, the study revealed reduced cerebral cortical volume and reduced entorhinal cortex thickness in dementia patients with poor sleep, an early feature of Alzheimer’s disease.

## Supplemental Material

sj-pdf-1-jcb-10.1177_0271678X221090747 - Supplemental material for Altered glymphatic enhancement of cerebrospinal fluid tracer in individuals with chronic poor sleep quality Click here for additional data file. Supplemental material, sj-pdf-1-jcb-10.1177_0271678X221090747 for Altered glymphatic enhancement of cerebrospinal fluid tracer in individuals with chronic poor sleep quality by Per Kristian Eide, Are Hugo Pripp, Benedikte Berge, Harald Hrubos-Strøm, Geir Ringstad and Lars Magnus Valnes in Journal of Cerebral Blood Flow & Metabolism
