# Supplementary material for: Altered glymphatic enhancement of cerebrospinal fluid tracer in individuals with chronic poor sleep quality
Source: J Cereb Blood Flow Metab. 2022 Mar 29;42(9):1676–92. doi: 10.1177/0271678X221090747 (PMC9441729; doi:10.1177/0271678X221090747)
Supplement: sj-pdf-1-jcb-10.1177_0271678X221090747 - Supplemental material for Altered glymphatic enhancement of cerebrospinal fluid tracer in individuals with chronic poor sleep quality [file sj-pdf-1-jcb-10.1177_0271678X221090747.pdf]

Supplementary material

Altered glymphatic enhancement of cerebrospinal fluid tracer in individuals with chronic poor sleep quality

Per Kristian Eide[1,2*], Are Hugo Pripp[3,4], Benedikte Berge[5], Harald Hrubos-Strøm[1,6] Geir Ringstad[7], Lars Magnus Valnes[2]

[1]Institute of Clinical Medicine, Faculty of Medicine, University of Oslo, Oslo, Norway.
[2]Department of Neurosurgery, Oslo University Hospital – Rikshospitalet, Oslo, Norway.
[3]Oslo Centre of Biostatistics and Epidemiology, Research Support Services, Oslo University Hospital, Oslo, Norway.
[4]Faculty of Health Sciences, Oslo Metropolitan University, Oslo
[5]Faculty of Medicine, University of Oslo, Oslo, Norway.
[6]Department of Otorhinolaryngology, surgical division, Akershus University Hospital.
[7]Department of Radiology, Oslo University Hospital- Rikshospitalet, Oslo, Norway.

Corresponding author:
Professor Per Kristian Eide, MD PhD
Department of Neurosurgery
Oslo University Hospital - Rikshospitalet
Pb 4950 Nydalen,
Phone: +47 91649419
Fax: +47-23074310
N-0424 Oslo, Norway
p.k.eide@medisin.uio.no

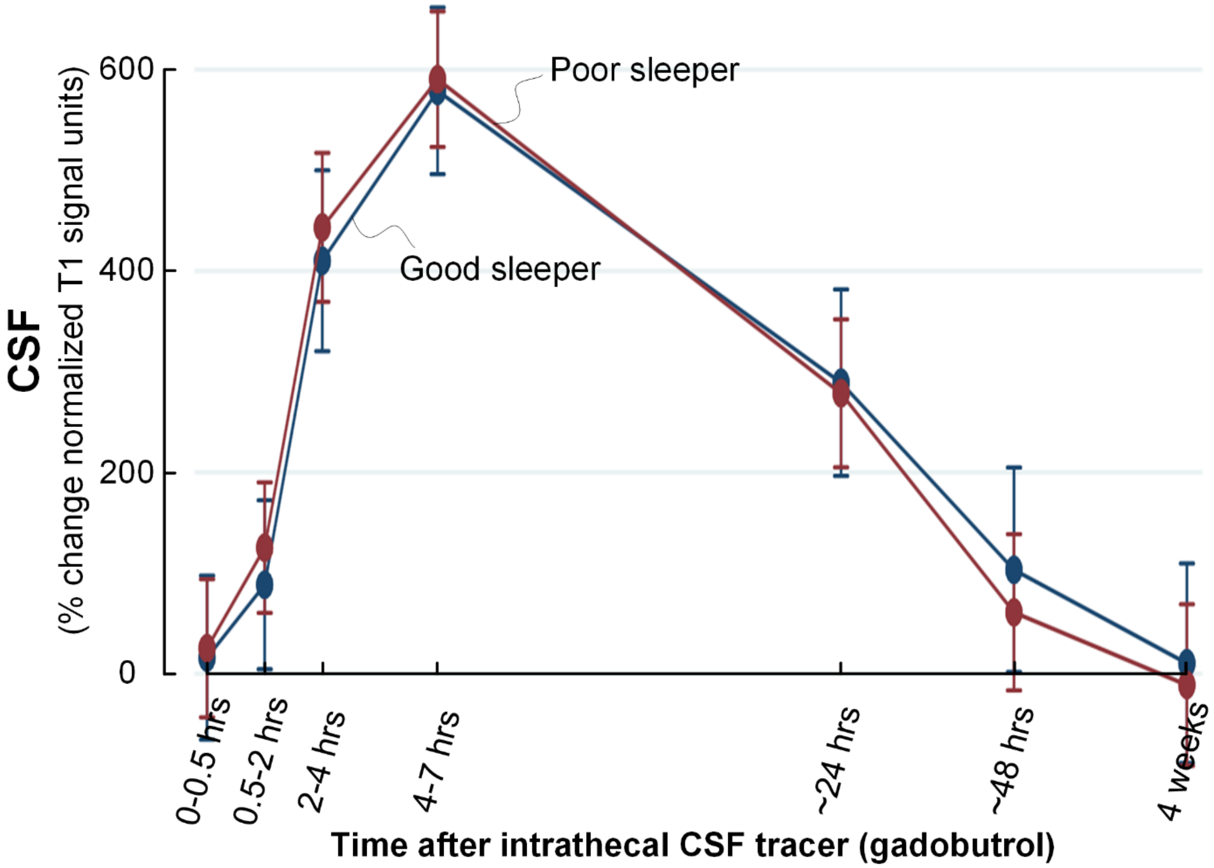

Supplementary Figure 1. Increased enrichment of CSF tracer within cerebrospinal fluid of individuals of cohort #1 with good or poor sleep quality. For patient cohort #1 with no diagnosed dementia disease, trend plots of percentage change in normalized T1 signal units from baseline are presented for cerebrospinal fluid as defined by FreeSurfer software (velum interpositum). Significant differences between the Poor sleepers (red line) and Good sleepers (blue line) are indicated as follows: *P<0.05, **P<0.01, ***P<0.001 (mixed model analysis; error bars: 95% confidence intervals).

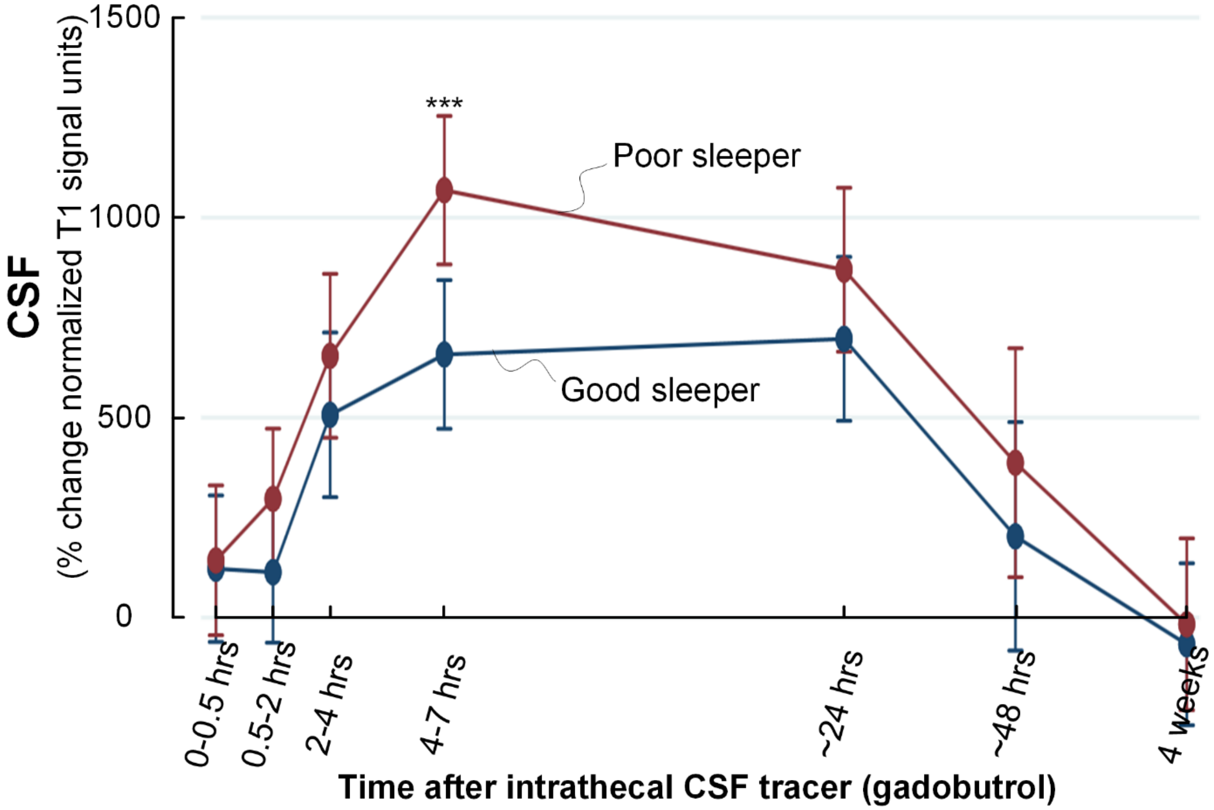

Supplementary Figure 2. Increased CSF tracer levels within cerebrospinal fluid of individuals of cohort #2 with good or poor sleep quality. For patient cohort #2 with the dementia subtype iNPH, trend plots of percentage change in normalized T1 signal units from baseline are presented for cerebrospinal fluid as defined by FreeSurfer software (velum interpositum). Significant differences between the Poor sleepers (red line) and Good sleepers (blue line) are indicated as follows: *$P<0.05$, **$P<0.01$, ***$P<0.001$ (mixed model analysis; error bars: 95% confidence intervals).

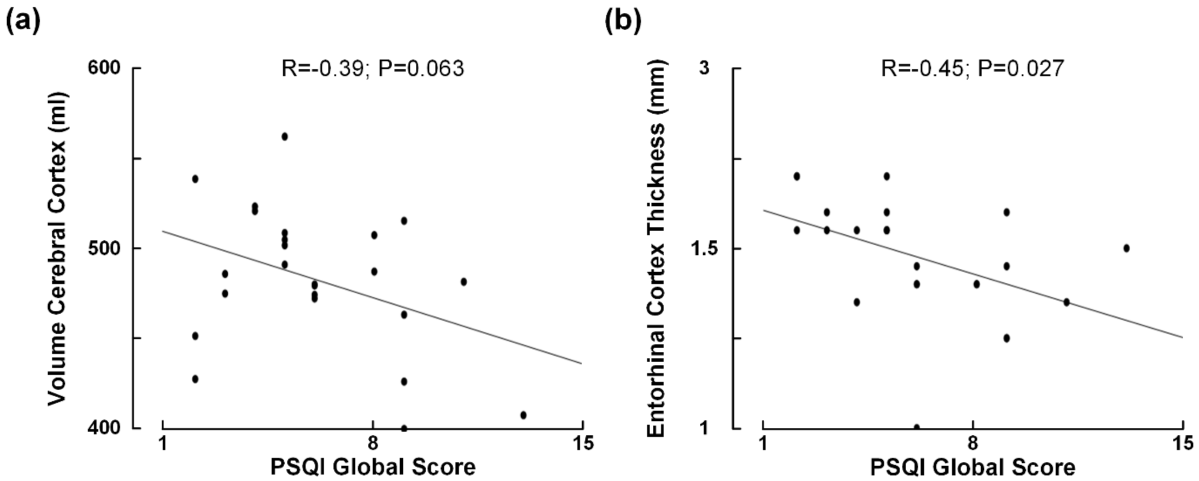

Supplementary Figure 3. Correlations between PSQI global score and volume of cerebral cortex in cohort #2. The Pearson correlation coefficient was non-significant between PSQI global score and volume of cerebral cortex (a), but significant between PSQI global score and entorhinal cortex thickness (b). Each scatter plot presents the fit line and the Pearson correlation coefficient (R) with P-value. PSQI: Pittsburgh Sleep Quality Index.

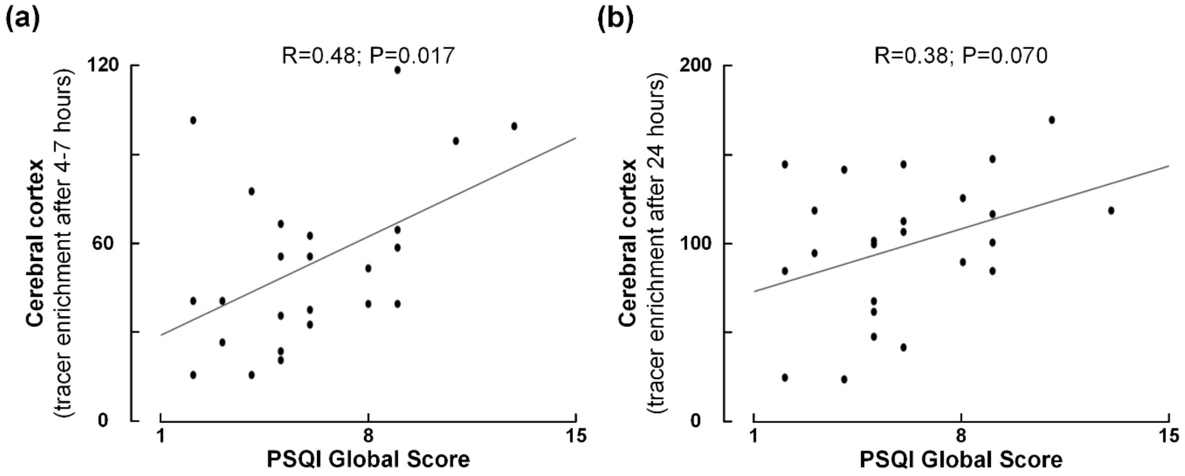

Supplementary Figure 4. Correlations between PSQI global score and tracer enrichment in cerebral cortex of cohort #2. The Pearson correlation coefficient was significant between PSQI global score and tracer enrichment after 4-7 hours in cerebral cortex (a), but non-significant between PSQI global score and tracer enrichment after 24 hours in cerebral cortex (b). Tracer enrichment refers to percentage change in normalized T1 signal units. Each scatter plot presents the fit line and the Pearson correlation coefficient (R) with P-value. PSQI: Pittsburgh Sleep Quality Index.

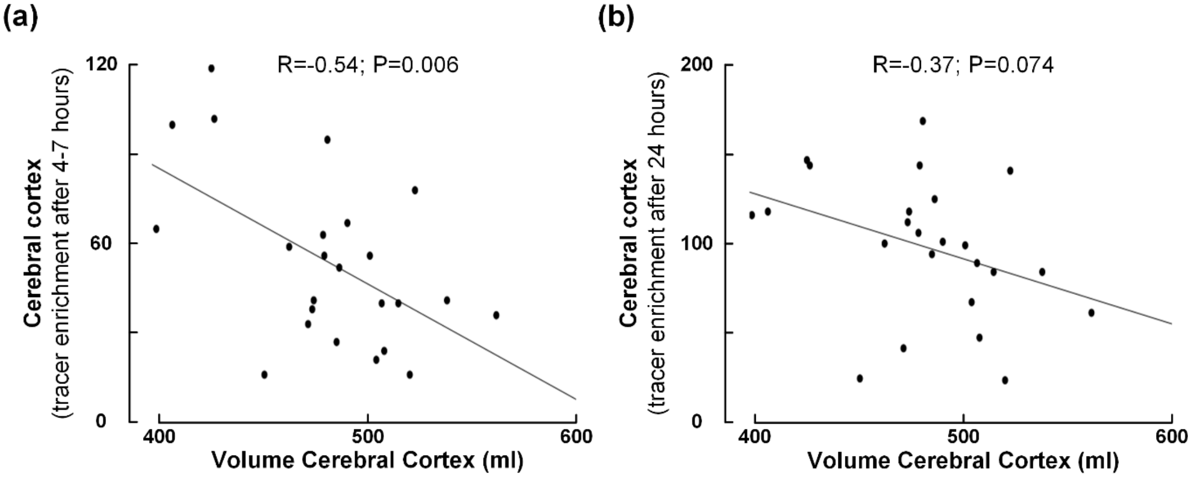

Supplementary Figure 5. Correlations between volume of cerebral cortex and tracer enrichment in cerebral cortex of cohort #2. The Pearson correlation coefficient was significant between volume of cerebral cortex and tracer enrichment after 4-7 hours in cerebral cortex (a), but non-significant between volume of cerebral cortex and tracer enrichment after 24 hours in cerebral cortex (b). Tracer enrichment refers to percentage change in normalized T1 signal units. Each scatter plot presents the fit line and the Pearson correlation coefficient (R) with P-value.
